# Visualizing and Quantifying Longitudinal Changes in Verbal Fluency Using Recurrence Plots

**DOI:** 10.3389/fnagi.2022.810799

**Published:** 2022-07-29

**Authors:** Samira A. Maboudian, Ming Hsu, Zhihao Zhang

**Affiliations:** ^1^Helen Wills Neuroscience Institute, University of California, Berkeley, Berkeley, CA, United States; ^2^Haas School of Business, University of California, Berkeley, Berkeley, CA, United States; ^3^Social Science Matrix, University of California, Berkeley, Berkeley, CA, United States; ^4^Darden School of Business, University of Virginia, Charlottesville, VA, United States

**Keywords:** recurrence plots, recall data, data visualization, Alzheimer’s disease, verbal fluency

## Abstract

The verbal fluency task, where participants name as many instances of a specific semantic or phonemic category as possible in a certain time limit, is widely used to probe language and memory retrieval functions in research and clinical settings. More recently, interests in using longitudinal observations in verbal fluency to examine changes over the lifespan have grown, in part due to the increasing availability of such datasets, yet quantitative methods for comparing repeated measures of verbal fluency responses remain scarce. As a result, existing studies tend to focus only on the number of unique words produced and how this metric changes over time, overlooking changes in other important features in the data, such as the identity of the words and the order in which they are produced. Here, we provide an example of how the literature of recurrence analysis, which aims to visualize and analyze non-linear time series, may present useful visualization and analytical approaches for this problem. Drawing on this literature, we introduce a novel metric (the “distance from diagonal,” or DfD) to quantify semantic fluency data that incorporates analysis of the sequence order and changes between two lists. As a demonstration, we apply these methods to a longitudinal dataset of semantic fluency in people with Alzheimer’s disease and age-matched controls. We show that DfD differs significantly between healthy controls and Alzheimer’s disease patients, and that it complements common existing metrics in diagnostic prediction. Our visualization method also allows incorporation of other less common metrics—including the order that words are recalled, repetitions of words within a list, and out-of-category intrusions. Additionally, we show that these plots can be used to visualize and compare aggregate recall data at the group level. These methods can improve understanding of verbal fluency deficits observed in various neuropsychiatric and neurological disorders.

## Introduction

Verbal fluency tasks, where participants are asked to produce as many instances of a specific semantic or phonemic category as possible in a short period of time, are commonly used to test a variety of cognitive and linguistic abilities, such as lexical knowledge, processing speed, verbal working memory, and executive functions (Troyer et al., 1997; Rosen, 1980; Shao et al., 2014).

In these tasks the experimental outputs consist of lists of categorical variables or items (words) produced in a certain order, an example of categorical time series data. Despite the multi-faceted nature of such data that may reflect distinct cognitive processes underlying task performance, in existing research participant performance is often scored in a manner that focuses on one particular characteristic of interest, most commonly the number of words produced (Ardila and Bernal, 2006). Similarly, while interest in comparing longitudinal observations of verbal fluency in aging and disease has increased as fluency tasks are commonly included in a battery of neuropsychological tasks administered in clinic visits over years, quantifying change over time is also frequently restricted to comparing the number of words produced between the generated lists (i.e., comparing the list lengths).

Quantifying only the total number of unique words generated has known limitations, despite its clinical significance (Henry et al., 2004; Cullen et al., 2007; Clark et al., 2009). It only captures a thin slice of the information available in fluency responses. Most prominently, it does not take into account changes in the identity of the items (what words they are); it doesn’t include alterations in the order items are produced, repeated items, items from the wrong category, or the subcategories items belong to. Therefore, changes in these features in verbal fluency performance over time and how they may be related to aging and disease development may have been under-appreciated, despite a growing volume of literature documenting differences in these features across populations in cross-sectional data. For example, a recent study reports differences in the correlation between the order of items recalled in a fluency list and various semantic features in old vs. young adults (De Marco et al., 2021). Repetitions of the same item within a list have been correlated with Alzheimer’s disease (AD) (Pekkala et al., 2008; McDowd et al., 2011) and frontotemporal dementia (Rascovsky et al., 2007). Intrusion errors involving incorrect words (e.g., “chair” within the semantic category “animals”) are also not considered in the traditional score and have been associated with AD (McDowd et al., 2011), semantic aphasia (Rogers et al., 2015), and schizophrenia (Allen et al., 1993). Differences in the clustering of words into semantic and phonemic categories have been associated with various disorders including mild cognitive impairment (MCI) and AD (Mueller et al., 2015; Pakhomov et al., 2016), as well as Huntington’s Disease (HD) (Ho et al., 2002). While longitudinal analysis of verbal fluency remains rare, changes in the repetition density and in the semantic relatedness of words chosen in fluency lists have been associated with AD and MCI (Pakhomov et al., 2016), while changes in the number of words produced, the number of repetitions, and phonemic clustering have been reported in HD (Ho et al., 2002).

These additional methods of comparing changes in fluency performance beyond the traditional length metric are not widely used by clinicians, potentially because they are more difficult to examine from raw responses than the number of items produced (Table 1). A significant challenge when comparing changes between lists is the multiple dimensions of dissimilarity that are hard to quantify and visualize—beyond the number of items produced, the order of words recalled and the words themselves may be changing as well. Here we sought to overcome these challenges by introducing a new way to visualize and quantify fluency data that incorporates analysis of the sequence order and changes between multiple lists. First, we describe a method to visualize these changes both at the individual and at the group levels, drawing from recurrence analysis methods. These visualizations emphasize salient aspects of fluency data, including changes in word ordering between lists, which have not been commonly quantified in this data (Table 1). Expanding upon this feature, we introduce a novel metric to quantify changes in the order of items recalled in fluency lists, termed the “distance from diagonal,” or DfD. The visualization and the metric derived from it complement each other, in a similar way as a correlation coefficient quantifies the strength of the relationship between two variables, while a scatterplot can demonstrate that relationship as well as providing insight into the shape (linearity or non-linearity) of the relationship and/or the presence of outliers (Anscombe, 1973).

**TABLE 1:** Clinically relevant features of semantic fluency data, and their incorporation into our visualization method.

Feature	Example	Clinical relevance	Visualization
**Length** of sequence: number of unique items	*Cat, dog, pig, cow, dog, duck, zebra, elephant, lion, shark, turtle* (length = 10)	Shorter lists associated with aging, mild cognitive impairment and AD (McDowd et al., 2011; Mueller et al., 2015; Taler et al., 2019)	Number of unique items on each axis

**Changes in length**	*Cat, dog, pig* vs. *Cat, dog, pig, cow, duck, zebra, elephant, lion, shark, turtle*	Decreases in length associated with normal aging (Taler et al., 2019), development and progression of MCI and AD (Mueller et al., 2015; Pakhomov et al., 2016), and Huntington’s disease (Ho et al., 2002)	Changes in aspect ratio: if sequences differ in length, the plot is rectangular instead of square

**Repetitions** (perseverations)	*Cat*, ***dog****, pig, cow*, ***dog****, duck, zebra, elephant, lion, shark, turtle*	Associated with AD (Pekkala et al., 2008; McDowd et al., 2011), and frontotemporal dementia (Rascovsky et al., 2007)	Points on the plot in the same row or column, further highlighted by a green arrow

**Intrusions** (out-of-category items)	*Cat, dog, pig, cow*, ***chair****, duck, zebra, elephant, lion, shark, turtle*	Associated with AD (McDowd et al., 2011), and schizophrenia (Allen et al., 1993)	Point highlighted by a red “x”

**Changes in order of items**	*Cat, dog, pig, cow, duck, zebra* vs. *Pig, dog, cat, cow, zebra, duck*	Correlation between recall order of words and their semantic features changes with age (De Marco et al., 2021); differences in clustering and switching emerge with aging (Troyer et al., 1997), and with MCI or AD (Fagundo et al., 2008)	Proximity of points to the diagonal (quantified by DfD score)

We employ semantic fluency as a usage case for this quantification and visualization method, and we show using an empirical dataset that these methods highlight multiple important features of semantic fluency performance, such as changes in item ordering between sequences. We show that the DfD metric quantifies changes in the order of items between sequences, which is found to differ significantly between healthy control and Alzheimer’s patients for semantic fluency. Individual participant plots facilitate tracking the progression of a participant’s performance over time on this task by easily and interactively demonstrating changes between the sequences. In addition, this visualization method can be extended to the group level to demonstrate differences in the patterns of recall sequences that are made by various groups (e.g., patients with AD or healthy controls).

## Methods

### Fluency Dataset and Participant Characteristics

For fluency data, we used a published dataset of longitudinal semantic fluency data from the University of California San Diego Shiley-Marcos Alzheimer’s Disease Research Center (UCSD ADRC) available at https://osf.io/j6qea/ (Zemla and Austerweil, 2019). This dataset contains semantic fluency lists for the category “animals” collected between 1985 and 2016 from 139 individuals (60% female). For this semantic fluency task, participants were given 60 seconds to name animals aloud, which were written down in real time by a researcher and later transcribed.

Each participant was tested approximately once per year during their involvement in the study and was given the fluency test as part of a larger set of tasks (Zemla and Austerweil, 2019). There are 20 conditions represented in the sample, but we restricted our analyses to participants considered healthy control (HC) or Probable Alzheimer’s Disease (ProbAD). Clinical diagnosis was based on the National Institute of Neurological and Communicative Disorders and Stroke-Alzheimer’s Disease and Related Disorders Association (NINCDS-ADRDA) scale, assessed at each visit. While age is not included in the transcribed dataset, as reported previously the mean age at first visit (used in these analyses) across all participants was 71.4 years (Zemla and Austerweil, 2019).

Within the dataset, 97 participants were considered HC and 61 were considered ProbAD at the time of data collection, but these groups are not mutually exclusive; 19 participants transitioned from one group to another at some point between 1985 and 2016. There are 1,167 total fluency lists from these participants: 785 from HC participants, 282 from ProbAD participants, and 100 from participants with other diagnoses. Because pairs of lists are required for the visualization, participants with fewer than two lists were excluded. To ensure sample independence, for statistical analysis only the first pair of lists for each participant was used, and the 19 participants in both the HC and ProbAD groups were also excluded. This resulted in a sample of 77 HC and 40 ProbAD participants with two fluency lists each. Within this group used for analyses, each participant had an average of 8 data timepoints (minimum of 2, maximum of 26), of which only the first pair was used to minimize repetition effects on task performance and ensure sample independence. This sample had 69 women (58.97%), consistent with the full dataset. As expected, the groups differed by Mini-Mental State Exam (MMSE) score, which was lower for the ProbAD group (*M* = 23.81, *SD* = 3.30) compared to HC (*M* = 29.04, *SD* = 1.87), *t*(52.29) = 9.25, *p* < 0.0001.

### Statistical Testing

Group differences across metrics—DfD score; number of words produced, i.e., list length; proportion of items in a list that are repeats; number of intrusions; and MMSE score—were compared using *t*-tests implemented in Python using Researchpy 0.1.9.

Logistic regression was used to determine the utility of including the DfD score above and beyond other metrics. Logistic regression was implemented using Statsmodels 0.12.2. Logistic regression models included common metrics used to quantify fluency (list length, repetitions, and intrusions), as well as a measure of change of an established metric (change in list length between two lists), and our DfD score. All metrics were z-scored. All models included all main effects and interaction terms between predictors (2-way to *n*-way). Pairs of models with and without the DfD score and its corresponding interactions were compared to assess its added value. Metrics included in each model and model comparisons are outlined in Table 2. Only models that converged are described in the table—models with both intrusions and repetitions were also run but did not converge. All models were also run with sex as an additional factor, but sex was not a significant predictor in any model and the overall model comparison results remained the same as indicated (Table 2).

**TABLE 2:** Regression results.

	DV: *Diagnosis (HC or ProbAD)*									
	
IV	1	2	3	4	5	6	7	8	9	10
*Average list length*	✓	✓	✓	✓	✓	✓				
*Length difference*					✓	✓	✓	✓	✓	✓
*Average proportion of repetitions*	✓	✓			✓	✓	✓	✓		
*Number of intrusions*			✓	✓					✓	✓
*DfD score*		✓		✓		✓		✓		✓

df	3	7	3	7	7	15	3	7	3	7
LL	−31.833	−24.865	−28.141	−24.319	−30.148	−21.842	−56.554	−32.924	−71.794	−45.051

Model comparisons	1 vs. 2		3 vs. 4		5 vs. 6		7 vs. 8		9 vs. 10	
LR, df, p	13.936, 4,		7.644, 4,		16.612, 8,		47.260, 4,		53.486, 4,	
	*p* = 0.0075		*p* = 0.11		*p* = 0.034		*p* = 1.4 × 10^–9^		*p* = 6.7 × 10^–11^	

*LL, log likelihood; LR, likelihood ratio. For each model, all main effects and interactions are included. Model comparisons are based on including/excluding the main effect of and all interactions involving the DfD score.*

Models were compared using the likelihood-ratio (LR) chi-squared test. MMSE was not included in any models in order to restrict comparison to methods of quantifying the semantic fluency task specifically.

### Interactive Notebook and Code Availability

All coding, data cleaning, and data analysis was done using Python 3.7.3 (NumPy 1.16.2,^1^ pandas 0.24.2,^2^ SciPy 1.6.2,^3^ Researchpy 0.1.9,^4^ Statsmodels 0.12.2,^5^ Matplotlib 3.0.3,^6^ interactive plots made with Plotly 4.1.0^7^).

Using Google Colaboratory, we created an interactive notebook to demonstrate the visualization of these data, which can be accessed at the following link: https://colab.research.google. com / drive / 11uS kmbw WUZnGNzt V1ulUWQxZ2VnhWnwX?usp=sharing. The code used for analyses and to generate the figures in this manuscript can be found on GitHub,^8^ and can be executed at that link *via* Binder.^9^

## Results

### Visualizing Changes in Semantic Fluency Data Using Recurrence Plots

**FIGURE 1:**
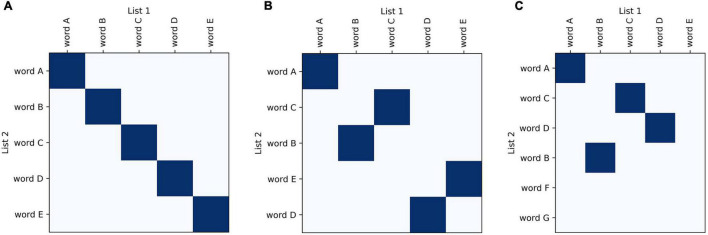
Basic features of recurrence plots. Basic visualization principles for recurrence plots of recall data: one sequence of words is plotted along one axis and another along the other axis; areas of similarity are shaded using dark blue points. In semantic fluency the two lists compared could be taken from two different time points when the task was performed to track progress, whereas for list learning the lists would be the actual list compared to the participant’s recalled sequence. Identical lists fill the diagonal **(A)**, whereas lists that are increasingly different look more scattered **(B,C)**.

Recurrence plots are commonly used in bioinformatics to compare nucleotide sequences (Gibbs and Mcintyre, 1970; Huang and Zhang, 2004; Cabanettes and Klopp, 2018). They have also been adapted for other fields, for example to analyze real-valued time series (Yankov et al., 2005) and large amounts of text or code (Church and Helfman, 1993). Drawing from these examples, we have developed a plotting method that allows visual and graphical comparison of semantic fluency sequences. Similar to other categorical time series data (such as nucleotide sequences), comparisons between fluency sequences can be visualized using similarity matrices. These plots are an attractive starting point for visualizing recall data because they do not employ a symbol-by-symbol representation and also do not require categories to have an inherent ordering. They visually highlight relevant aspects of recall data; one sequence of words can be plotted along the x-axis and the other along the y-axis with matching points shaded, allowing for visualization of changes between sequences such as changes in ordering and missing or added words (Figure 1).

**FIGURE 2:**
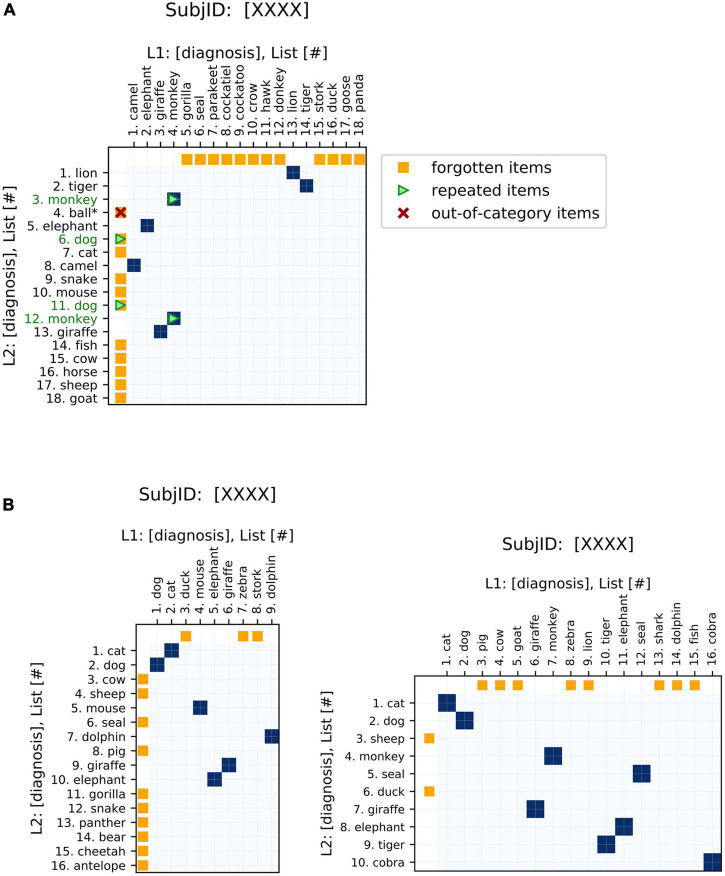
Basic features of semantic fluency visualizations. **(A)** The first sequence (chronologically) is plotted on the x-axis and the second on the y-axis. Areas of similarity are shaded in blue. Items present in one sequence but forgotten in the other are indicated by orange points on the corresponding axis, i.e., orange points on the x-axis indicate items present in the first (x-axis) list but missing from the second (y-axis) list. Repeated items are indicated by green arrows and green item labels. For semantic fluency data specifically, invalid (out-of-category) items, also referred to as intrusions, are indicated by a red “x” and asterisk. Repetitions and out-of-category items can occur as words present in both lists (blue points) or just in one list (orange), so these markers are overlaid on the appropriate color point. **(B)** Changes in sequence length are easily visualized *via* the aspect ratios of the plots. Square plots correspond to no change, landscape-oriented rectangles (right) correspond to the second sequence being shorter in length, and portrait-oriented rectangles (left) correspond to the second sequence being longer.

As one of the main strengths of these plots, changes in item ordering between sequences are highlighted. These plots emphasize the changes in both the identity and order of the words generated, and also conveniently represent unidimensional metrics of semantic fluency performance (see Table 1, ‘Visualization’ for a summary). The traditional fluency metric (the number of words produced) can be compared between the two sequences plotted based on the lengths of the axes. Changes in the number of words produced between the two sequences are demonstrated by changes in the dimensions of the plot (Figure 2). These plots allow for the comparison of fluency performance changes over multiple years, aiding the ability to monitor performance on the task over time.

In order to increase the relevance of the plots to analyzing recall data, we have added some additional features to the recurrence plots that are specific to this application. These elements emphasize aspects of interest to fluency data, such as repeated items or missing items between sequences (Figure 2). These highlighted features are clinically relevant to the analysis of semantic fluency data: differences in repetitions, out-of-category items, and changes in the length of the sequences produced have all been associated with AD (Table 1). Additional elements could be tailored to other specific applications.

**FIGURE 3:**
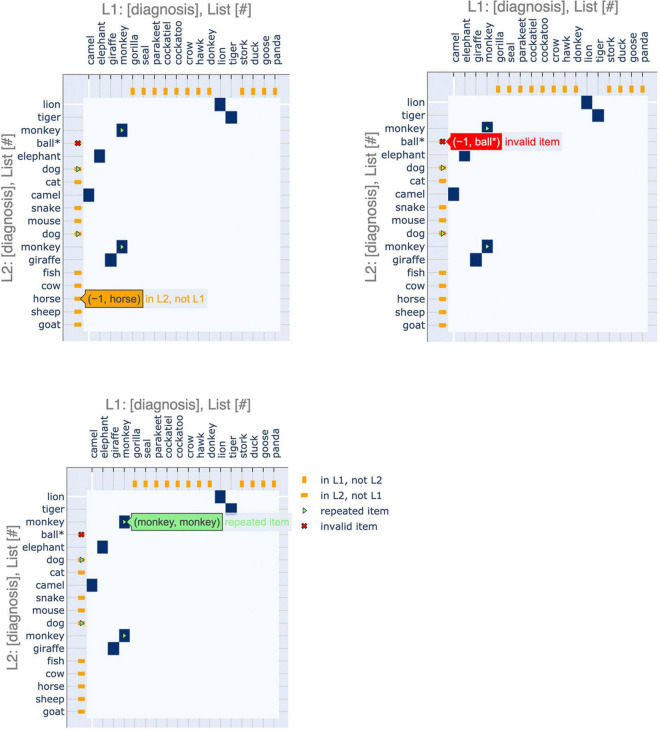
Interactive features of visualizations. The plots can be made interactive to facilitate usage. Hovering over a point shows the identity of the items that it corresponds to as well as salient features, such as repeated or invalid items.

Furthermore, we have incorporated interactive features to make the application more user-friendly, especially for clinical practitioners (Figure 3). These interactive displays facilitate navigating the plots and their features by providing tooltips that describe sequence items and any special attributes. These features can be accessed using the following Google Colaboratory notebook: https://colab.research.google.com/drive/11uSkmbwWUZnGNztV1ulUWQxZ2VnhWnwX?usp=sharing.

### Quantifying Changes in Semantic Fluency

With these recurrence plots, changes in item ordering between sequences stand out. Drawing from these visualizations, we developed the following metric to quantify the difference in the ordering of words produced between sequences, termed the “distance from diagonal,” or DfD:


$$D\,f\,D=\sum_{\left\{i,j\right\}}m_{i,j}*\left|i-j\right|,$$

where (i, j) are the indices of the array, and *mi*, *j* = 1 if the items on the two axes match and *m*_*i*, *j*_ = 0 otherwise. The DfD of a pair of sequences is therefore 0 if the sequences are a perfect match, and increases as the order of items is more scrambled. Lower DfD scores indicate sequences are closer to a perfect match with each other in the words produced and their ordering. For our analyses, the DfD, like the traditional metric of list length, is calculated using only the unique words produced (repetitions of words are removed).

Using this new metric, we find that patients with Probable AD (ProbAD) have a lower DfD score on average (*M* = 14.53, *SD* = 11.36) than healthy controls (HC) (*M* = 43.53, *SD* = 27.99), *t*(110.23) = 7.93, *p* < 0.0001. This finding demonstrates that patients with ProbAD tend to have more similar lists over time, since the DfD quantifies the similarity between a pair of lists (and lower is more similar). This result may occur in ProbAD because as their semantic networks shrink, patients with ProbAD tend to output the same few familiar items, while healthy controls have a larger, more intact network to choose from. This is consistent with previous reports that words selected earlier in semantic fluency lists tend to be those most frequently used in a given language, which are also those most preserved in AD (Tang-Wai and Graham, 2008; De Marco et al., 2021).

Next, we sought to examine if the DfD metric offers additional diagnostic value, above and beyond existing metrics including list length, repetitions, and intrusions. To this end, we first confirm that, as previously reported (McDowd et al., 2011; Mueller et al., 2015; Pakhomov et al., 2016), patients with ProbAD produce fewer correct words on average (*M* = 12.54, *SD* = 3.67) than HC (*M* = 19.55, *SD* = 3.35), *t*(73.06) = 10.09, *p* < 0.0001. Since patients with AD have been found to repeat items more within a list (Pekkala et al., 2008; McDowd et al., 2011), we also calculated the proportion of items in a list that are repeated (i.e., the number of repetitions in a list divided by the length of the list). We confirm that the average proportion of repetitions per list is higher for ProbAD patients (*M* = 0.090, *SD* = 0.086) than HC (*M* = 0.020, *SD* = 0.027), *t*(43.05) = −5.03, *p* < 0.0001. The number of intrusions is also higher for the ProbAD group (*M* = 0.40, *SD* = 0.71) than HC (*M* = 0.14, *SD* = 0.39), *t*(51.43) = −2.13, *p* = 0.038.

We then combined the novel DfD metric and the existing metrics in a series of logistic regression analyses to test if and to what extent the DfD metric adds diagnostic value. In these models we included existing metrics used to quantify fluency (list length: the average length of a pair of lists, repetitions: the average proportion of repetitions in the pair of lists, and intrusions: the number of intrusions in the pair) as well as our DfD score for the pair of lists. Consistent with our hypothesis, models predicting diagnosis that included the DfD score consistently outperformed models without it (Table 2, models 1–4), although in one case this difference does not reach significance (Table 2, models 3 and 4). In an additional set of comparisons, we included the difference between the lengths of the lists, to indicate if comparing changes in ordering over time (DfD score) adds diagnostic value even when considering changes in the classic list length metric over time. In this case the models that included the DfD score again consistently outperformed those that did not (Table 2, models 5–10).

### Visualizing Aggregate Comparisons of Fluency Performance

**FIGURE 4:**
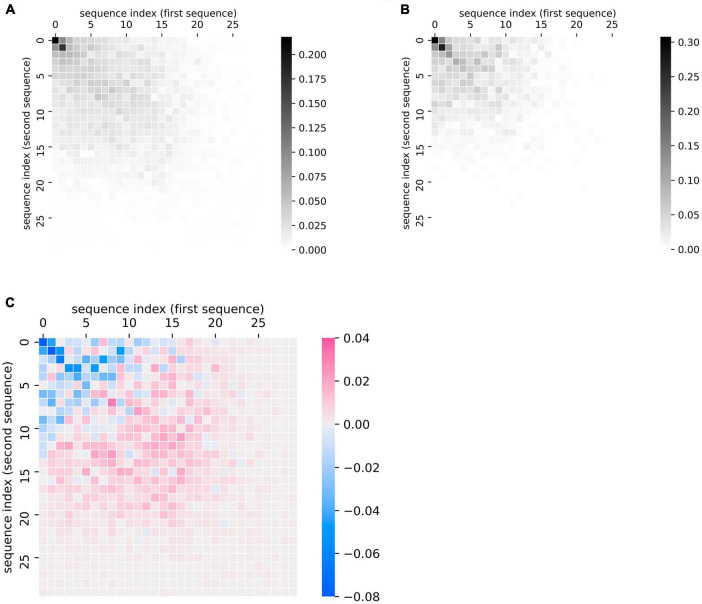
Visualizing group-level comparisons of fluency data. **(A)** Cumulative plots of every chronologically adjacent pair of sequences (i.e., a sequence from one clinic visit paired with one from the next) from the same participant from visits where the participant was diagnosed as healthy control. Individual similarity matrices (where 1 indicates a position of overlap between the sequences and 0 indicates no overlap) are added together and then scaled (by dividing by the total number of matrices or plots in each group). Scale bar thus indicates the proportion of matrices where the indicated point overlaps between a participant’s two lists. *n* = 97 participants, 724 pairs of sequences. **(B)** Cumulative plots of every chronologically adjacent pair of sequences from the same participant from visits where the participant was diagnosed as Probable AD. Individual similarity matrices are added together and then scaled as described above. *n* = 61 participants, 221 pairs of sequences. **(C)** Subtraction of the cumulative scaled plot of healthy control participants **(A)** minus the cumulative plot of Probable AD participants **(B)**. Scale bar indicates how much more likely a point is to belong to the overall control (pink) or ProbAD (blue) group pattern. Pink areas correspond to relatively stronger healthy control patterns (controls tend to have more lists that overlap in these areas) and blue colors correspond to stronger Probable AD patterns (ProbAD participants tend to have more lists that overlap in these areas).

This plotting method can also be applied at the group level to summarize group differences in the patterns of recall data, as quantified in the previous section. We demonstrate this application by comparing sequences made by patients with ProbAD or healthy control participants. Using the UCSD ADRC dataset described above, we generated plots of every chronologically adjacent pair of lists that belonged to the same participant; we then generated a cumulative heatmap of these plots by adding the similarity matrices together and scaling them (by dividing by the total number of matrices or plots in each group). We did so for all fluency sequences corresponding to healthy controls (Figure 4A) and separately for all sequences corresponding to ProbAD patients (Figure 4B).

This method allows for the visualization of general group patterns, e.g., in healthy participants and participants with ProbAD (Figures 4A,B). It also allows for more direct visual group comparisons: subtracting the plot of participants with ProbAD (Figure 4B) from the healthy controls plot (Figure 4A) generates a heatmap demonstrating group differences, which shows that responses for participants with ProbAD tend to be shorter and closer to the diagonal (Figure 4C). These results may assist users in better interpreting results from quantitative analysis of the group (such as those described in “Quantifying Changes in Semantic Fluency”), and corroborate the findings above that patients with ProbAD produce fewer correct words and have a lower DfD score on average, meaning their sequences tend to be more similar in order.

## Discussion

We introduce a novel metric for quantifying changes in recall data, the DfD, which emphasizes discrepancies in item ordering between sequences. We also illustrate a new way to visualize this kind of data that complements this metric, highlighting changes in item ordering, list length, and item identity between sequences that can be difficult to distinguish from raw responses or current metrics alone. We demonstrate a specific application of these methods to a type of recall data (semantic fluency data), and highlight a clinical application in the form of visualizing and quantifying changes in semantic fluency performance in Probable AD. We show that quantifying changes *via* the DfD metric provides additional diagnostic value beyond existing metrics. Furthermore, we show that these plots can be used to visualize and compare aggregate fluency performance (or other recall data) between groups to highlight group-level changes.

While we focus on applying these methods to verbal fluency tasks, studies of human memory and language make use of a variety of paradigms designed to engage various aspects of memory retrieval or language production: (i) cued recall tests, such as verbal fluency tasks (e.g., Nelson and McEvoy, 1979); (ii) free recall list-learning tests, where participants study a list of items on each trial and then are prompted to recall the items in any order (e.g., Cohen, 1963; d’Ydewalle, 1981; Nelson et al., 1982; Sadeh et al., 2018); and (iii) serial recall list-learning tests, where participants study a list of items and are asked to recall them in order (e.g., Klein et al., 2005; Chubala et al., 2019). Because of the common focus on comparing pairs of lists, the methods outlined here can also be easily adapted to list-learning tasks or other recall data and may provide additional insight into the quantification of these tasks. For example, it may be beneficial to quantify deviations in the ordering of recalled items from the original learned list in serial recall list-learning tasks, which could be accomplished with the DfD metric.

Given the challenges of visualizing categorical time series data and the specific constraints of recall data (Weiß, 2008), these recurrence plots are an appealing way to visualize this type of data because they do not require a symbol-by-symbol representation of each item or a natural ordering of categories. They also demonstrate relevant aspects of recall data and can be used to track changes in this data over time. However, visualizing nominal categorical time series data usually requires solutions tailored to a certain type of data and to the goals of analysis (Weiß, 2018). Therefore, these recurrence plots incorporate symbols and visualizations specific to analyzing recall data and thus may not necessarily be applicable to other kinds of categorical time series data without modification. This visualization method also does not provide very meaningful information for extremely short sequences or, in the case of applications to semantic fluency performance, for sequences that are extremely disorganized or unintelligible (such as those that may be produced by patients with aphasia).

As an additional limitation, one aspect of recall data in general and semantic fluency data in particular that is not explicitly captured by these quantifications or recurrence plots is the semantic relationships between words. Clustering words into semantic subcategories has been used to analyze semantic fluency performance; differences in the number of subcategories and the amount of switching between subcategories have been reported between young and elderly healthy adults (Troyer et al., 1997), and between healthy elderly adults and those with AD (Fagundo et al., 2008). Recurrence plots visualize sequence similarities, but since they do not use any form of symbol-by-symbol representation they do not visualize features of specific items (beyond sequence similarity) or semantic relationships between items. Future work incorporating a method of quantifying and visualizing semantic clustering for fluency data could further enhance its scope and practical appeal.

In terms of other avenues for future directions of this work, the use of network analysis and graph theory to capture network properties of human memory and recall is becoming increasingly popular (Lerner et al., 2009; Bertola et al., 2014). These methods can provide important insights into memory processes, but one hurdle is that the performance of these models is difficult to visualize. Similar methods to those described in this paper, such as the DfD metric and aggregate group-level recurrence plots, can be used to compare the performance of various network models by visualizing and quantifying their outputs. The DfD metric described in this paper is a straightforward quantification of the visual patterns demonstrated in these recurrence plots, but is not the only way to quantify patterns in this kind of data. Future work geared toward developing additional metrics could have important implications for improving the analysis of recall data and graph theory models.

More precise quantification and visualization of recall data can have other important scientific and clinical value. One clinical application of these methods that we demonstrate is for analyzing semantic fluency data in patients with AD. Certain features of fluency performance, such as the amount of repetitions in the list, increase with the severity of AD (Pekkala et al., 2008). It would be interesting to analyze these plots and metrics in a larger dataset with more patients who transition from being healthy control to a probable AD diagnosis (or who develop MCI and then probable AD) to see if these diagnostic transitions can be predicted earlier using these visualizations and the DfD metric than using the traditional fluency metric (the length of the list) alone. If so, these methods could provide clinicians with an additional way to track and visualize disease progression in MCI and AD.

Beyond AD, fluency performance is also used to evaluate other disorders. Semantic fluency scores correlate significantly with MMSE scores, making fluency tests attractive for clinical settings because they are short and easier to administer (Lopes et al., 2009). Less time or fewer words between repetitions in fluency suggests a retrieval error, as seen in aphasics, whereas more time or words between repetitions suggests a working memory deficit, as seen in patients with AD (Miozzo et al., 2013). Patients with schizophrenia show deficits in memory, attention, executive functioning, and psychomotor speed, and they also show deficits in fluency performance (van Beilen et al., 2004). Verbal fluency performance has also been used to analyze lexical processing and executive function in attention-deficit hyperactivity disorder (Takács et al., 2014). These quantification and visualization methods could thus be applied to verbal fluency analysis in these other clinical settings as well. These methods could also be applied to other types of recall data beyond semantic fluency data, such as performance on list learning paradigms, where the order of items recalled is crucial for analysis.

## Data Availability Statement

The original contributions presented in this study are included in the article/supplementary material, further inquiries can be directed to the corresponding author.

## Author Contributions

SM performed the coding and data analysis. ZZ and MH verified the analytical methods. All authors conceptualized and designed the research and discussed the results and wrote the final manuscript.

## Conflict of Interest

The authors declare that the research was conducted in the absence of any commercial or financial relationships that could be construed as a potential conflict of interest.

## Publisher’s Note

All claims expressed in this article are solely those of the authors and do not necessarily represent those of their affiliated organizations, or those of the publisher, the editors and the reviewers. Any product that may be evaluated in this article, or claim that may be made by its manufacturer, is not guaranteed or endorsed by the publisher.

## Footnotes

1
https://numpy.org/

2
https://pandas.pydata.org/

3
https://scipy.org

4
http://researchpy.readthedocs.io/

5
https://statsmodels.org

6
https://matplotlib.org/

7
https://pypi.org/project/plotly/

8
https://github.com/smaboudian/recurrence-analysis

9
https://mybinder.org/
